# ^1^H, ^13^C and ^15^N assignments of the four N-terminal domains of human fibrillin-1

**DOI:** 10.1007/s12104-012-9456-0

**Published:** 2012-12-23

**Authors:** David A. Yadin, Ian B. Robertson, Sacha A. Jensen, Penny A. Handford, Christina Redfield

**Affiliations:** Department of Biochemistry, University of Oxford, South Parks Road, Oxford, OX1 3QU UK

**Keywords:** Human fibrillin-1 N-terminus, NMR resonance assignments, Extracellular matrix, Microfibril assembly, Bone morphogenetic proteins

## Abstract

Fibrillins are extracellular, disulphide-rich glycoproteins that form 10–12 nm diameter microfibrils in connective tissues. They are found in the majority of higher animals, from jellyfish to humans. Fibrillin microfibrils confer properties of elasticity and strength on connective tissue and regulate growth factor availability in the extracellular matrix (ECM). Mutations in *FBN1*, the human gene encoding the fibrillin-1 isoform, are linked to several inherited connective tissue disorders. The fibrillin-1 N-terminus forms many functionally-important interactions, both with other fibrillin molecules and various ECM components. In particular, the first four domains, the fibrillin unique N-terminal (FUN) and three epidermal growth factor (EGF)-like domains (FUN-EGF3), are implicated in microfibril assembly and growth factor sequestration. The structure of these domains, which comprise 134 residues, is unknown. We have produced a recombinant fragment corresponding to this region of human fibrillin-1. Here, we report ^1^H, ^13^C and ^15^N resonance assignments of the FUN-EGF3 fragment. Assignments will facilitate structure determination, analysis of interdomain dynamics and the mapping of interaction surfaces.

## Biological context

Fibrillin microfibrils are essential structural and regulatory components of the extracellular matrix (ECM) in connective tissues. They are found in elastic tissues, such as the skin, blood vessels and lungs, as part of elastic fibres, in which they surround an amorphous network of elastin (Sakai et al. 1986). Microfibrils are also present in non-elastic connective tissues such as the ciliary zonules of the eye and kidney glomerulus, which do not contain elastin. In both cases, microfibrils are important for strength and elasticity. Furthermore, they regulate the availability of transforming growth factor-beta (TGFβ) family signalling molecules through sequestration of their latent forms (Ramirez and Rifkin 2009). When extracted from tissue and visualised by electron or atomic force microscopy, microfibrils have a diameter of 10–12 nm and a beaded filament structure, with an average distance of ~56 nm between the beads (Sakai et al. 1991, Hanssen et al. 1998).

**Fig. 1:**
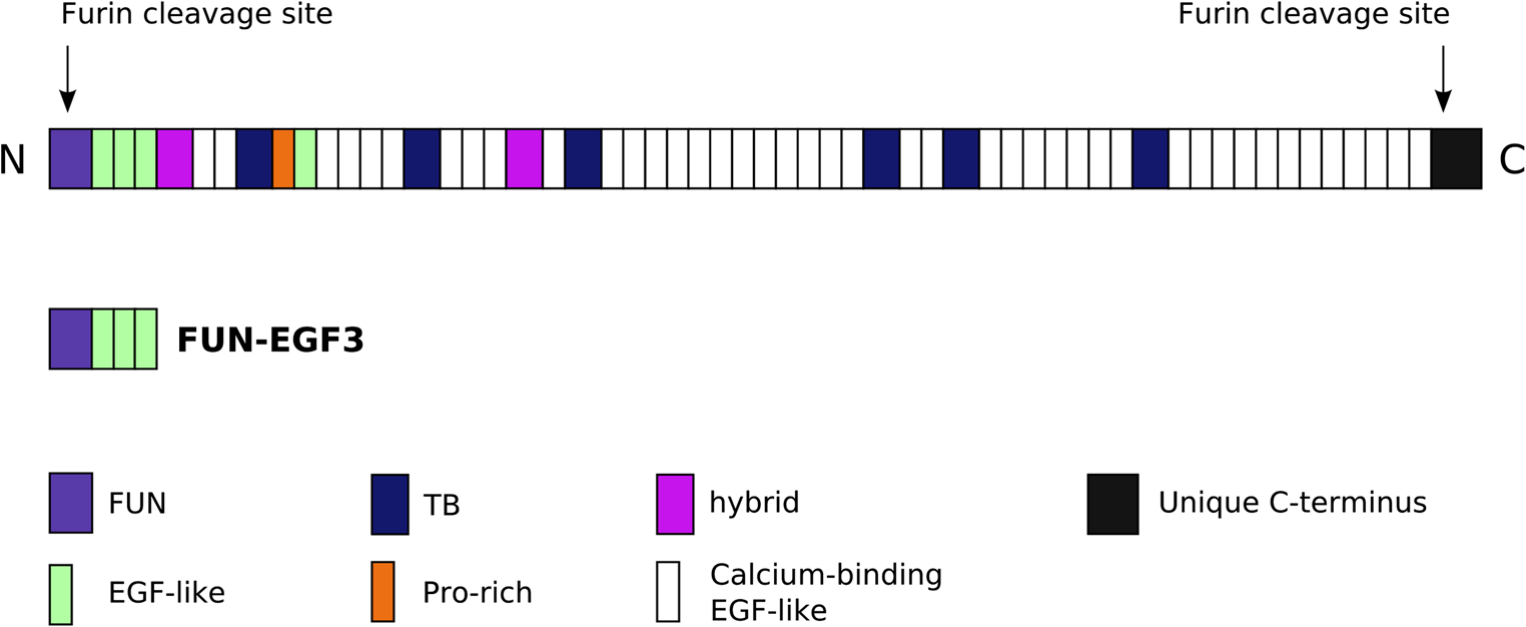
Domain organisation of human fibrillin-1. Fibrillins have two furin-cleavage sites—one at the N-terminus preceding the FUN domain and one in the unique C-terminal domain. The fragment under study (FUN-EGF3), which begins after the first furin recognition site, is *highlighted*

Fibrillins, the major structural components of microfibrils, are disulphide-rich glycoproteins found in most eumetazoa (Robertson et al. 2011). Their domain organisation is modular and highly conserved, with a high abundance of calcium-binding epidermal growth factor-like (cbEGF) domains, as well as several non-calcium EGF domains (Fig. 1). Arrays of cbEGF domains are interspersed by TGFβ binding-like (TB) and hybrid (hyb) domains, as well as a proline- or glycine-rich region, depending on the isoform. The structures of many of these domain types have been determined by X-ray crystallography and NMR spectroscopy (Knott et al. 1996, Yuan et al. 1997, Lee et al. 2004, Jensen et al. 2009). However, detailed structural information about the N- and C-terminal regions, including the fibrillin unique N-terminal (FUN) domain, is still lacking. High-resolution structures are important for understanding how fibrillin monomers assemble into microfibrils, the arrangements they form in microfibrils, their interactions with other ECM components and the effects of disease-causing mutations. Several inherited connective tissue disorders are linked to mutations in the human *FBN1* gene, which encodes fibrillin-1: for example, Marfan syndrome (Lee et al. 1991), stiff skin syndrome (Loeys et al. 2010), acromicric and geleophysic dysplasias (Le Goff et al. 2011) and Weill-Marchesani syndrome (Faivre et al. 2003).

The fibrillin N-terminus is functionally versatile, interacting with many ECM components. It has been suggested that an important step in microfibril assembly is the interaction between the N- and C-terminal regions of fibrillin monomers. Several studies have demonstrated such interactions (Lin et al. 2002, Marson et al. 2005, Hubmacher et al. 2008). The minimal regions needed for the N–C terminal interaction have been identified: the four N-terminal domains (FUN-EGF3), starting after the furin proteolytic cleavage site, and the last three cbEGF domains (cbEGF41–cbEGF43) at the C-terminus (El-Hallous et al. 2007, Hubmacher et al. 2008). The FUN-EGF3 region is also a minimal interaction site for the prodomains of bone morphogenetic proteins-5 (Sengle et al. 2011) and -7 (Sengle et al. 2008). As a first step towards structural analysis of this region, a recombinant FUN-EGF3 fragment was produced. ^1^H, ^13^C and ^15^N resonance assignments for the protein are reported here.

## Methods and experiments

### Protein expression, purification and refolding

Protein expression and purification was carried out as described previously (Knott et al. 1996) with modifications. *Escherichia coli* BL21 cells, also containing the pREP4 plasmid (Qiagen) encoding the lacI repressor, were transformed with the pQE-30 expression vector (Qiagen) containing the human fibrillin-1 FUN-EGF3 sequence. The native fibrillin sequence started at residue R45 (numbered according to Pereira et al. 1993), the first residue after the furin proteolytic cleavage site, and finished at Q178, the residue after the last cysteine in the EGF3 domain. An N-terminal His_6_ tag was present for purification, followed by a factor Xa protease recognition site (I-E-G-R) to allow His_6_ tag removal and an S–A spacer before the start of the FUN-EGF3 sequence. ^15^N/^13^C-double labelled protein was produced by growing cells in M9 medium containing 0.1 % (w/v) ^15^NH_4_Cl and 0.5 % (w/v) ^13^C-glucose (Cambridge Isotope Laboratories), with the addition of 100 μg/ml ampicillin and 25 μg/ml kanamycin. 50 ml of starter culture, grown in labelled medium at 37 °C for ~18 h, was added to 0.6 l of medium. Cells were grown to OD_600_ ~0.8 and expression was induced with isopropyl-β-d-thiogalactopyranoside (IPTG) at a final concentration of 2 mM. Cells were incubated at 28 °C for at least 6 h, harvested by centrifugation and stored at −80 °C until protein purification.

Cells were initially lysed in buffer containing 50 mM sodium phosphate pH 7.4, 6 M guanidinium chloride and 5 mM 2-mercaptoethanol (lysis buffer). Insoluble material was pelleted by ultracentrifugation (40,000 rpm; Beckmann 60Ti rotor; 25 °C; 30 min). The insoluble material was resuspended in lysis buffer. 2-mercaptoethanol was added to the suspension (concentration increase of 0.3 mM) and the pH was raised to ~9.0 with the addition of NaOH, followed by vigorous mixing by vortexing. The pH was then lowered to pH ~7.4 using HCl and the solution was centrifuged again. Cleared lysate was loaded onto fast flow chelating Sepharose (GE Healthcare) pre-loaded with Ni^2+^ and pre-equilibrated with lysis buffer. The column was then washed extensively with lysis buffer. Protein was eluted from the column in lysis buffer also containing 50 mM ethylenediaminetetraacetic acid (EDTA) and 100 mM dithiothreitol (DTT). Tris–HCl pH 8.3 was added to the eluted protein at a final concentration of 100 mM and additional DTT to final concentration of 200 mM. Following incubation at room temperature for 1 h, the mixture was adjusted to pH ~3.0 and dialysed overnight against 0.1 % (v/v) trifluoracetic acid (TFA). Protein was then desalted by reverse phase high performance liquid chromatography (HPLC).

Purified, reduced protein was refolded under the following conditions: 100 mM Tris–HCl pH 8.3, 3 mM l-cysteine, 0.3 mM l-cystine, 50 % (v/v) glycerol, 0.2 mg/ml protein at 4 °C for ~72 h. The refolding mixture was acidified to pH ~3.0 and dialysed against 0.1 % (v/v) TFA for at least 5 h. Protein was then concentrated by ultrafiltration and purified by HPLC. The His_6_ tag was removed through treatment with factor Xa (Novagen) under the conditions: 20 mM 2-(N-Morpholino) ethanesulfonic acid pH 5.8, 100 mM NaCl, ~4 mg/ml protein, 1 unit factor Xa/mg protein. Protein was further purified by cation exchange fast liquid protein chromatography and HPLC. The final product was analysed by denaturing SDS-PAGE in the presence or absence of 5 % (v/v) 2-mercaptoethanol, as well as by electrospray ionisation mass spectrometry (observed mass of unlabelled protein is 14,082 Da).

### NMR spectroscopy

Experiments for assignment were performed using ^15^N-single or ^15^N/^13^C-double labelled FUN-EGF3 at a concentration of 0.5–1.5 mM in 5 % D_2_O/95 % H_2_O (v/v) at pH 5.4. NMR experiments were carried out at 298 K on three different spectrometers: home-built spectrometers with ^1^H-operating frequencies of 600 MHz or 750 MHz, a triple-resonance probe and GE/Omega console, or a Bruker Avance 500 MHz spectrometer with a Cryoplatform, equipped with a TCI CryoProbe. Initial sequential assignments were obtained with data from 3D ^15^N-edited TOCSY-HSQC, NOESY-HSQC and HSQC-NOESY-HSQC experiments acquired using ^15^N-labelled protein. Assignments were confirmed and extended using 3D HNCA, (H)CC(CO)NH, HNCO and HN(CA)CO experiments with ^15^N/^13^C-double labelled FUN-EGF3. Side-chain assignments were obtained using the above experiments, as well as 3D HCCH-TOCSY, 3D ^13^C-edited NOESY-HSQC, 2D ^1^H-^13^C(aromatic) HSQC, 2D ^1^H NOESY, 2D ^1^H DQF-COSY and 2D ^1^H TOCSY. NMR data were processed using NMRPipe (Delaglio et al. 1995) and analysed using CcpNmr Analysis (Vranken et al. 2005).

## Assignments and data deposition

**Fig. 2:**
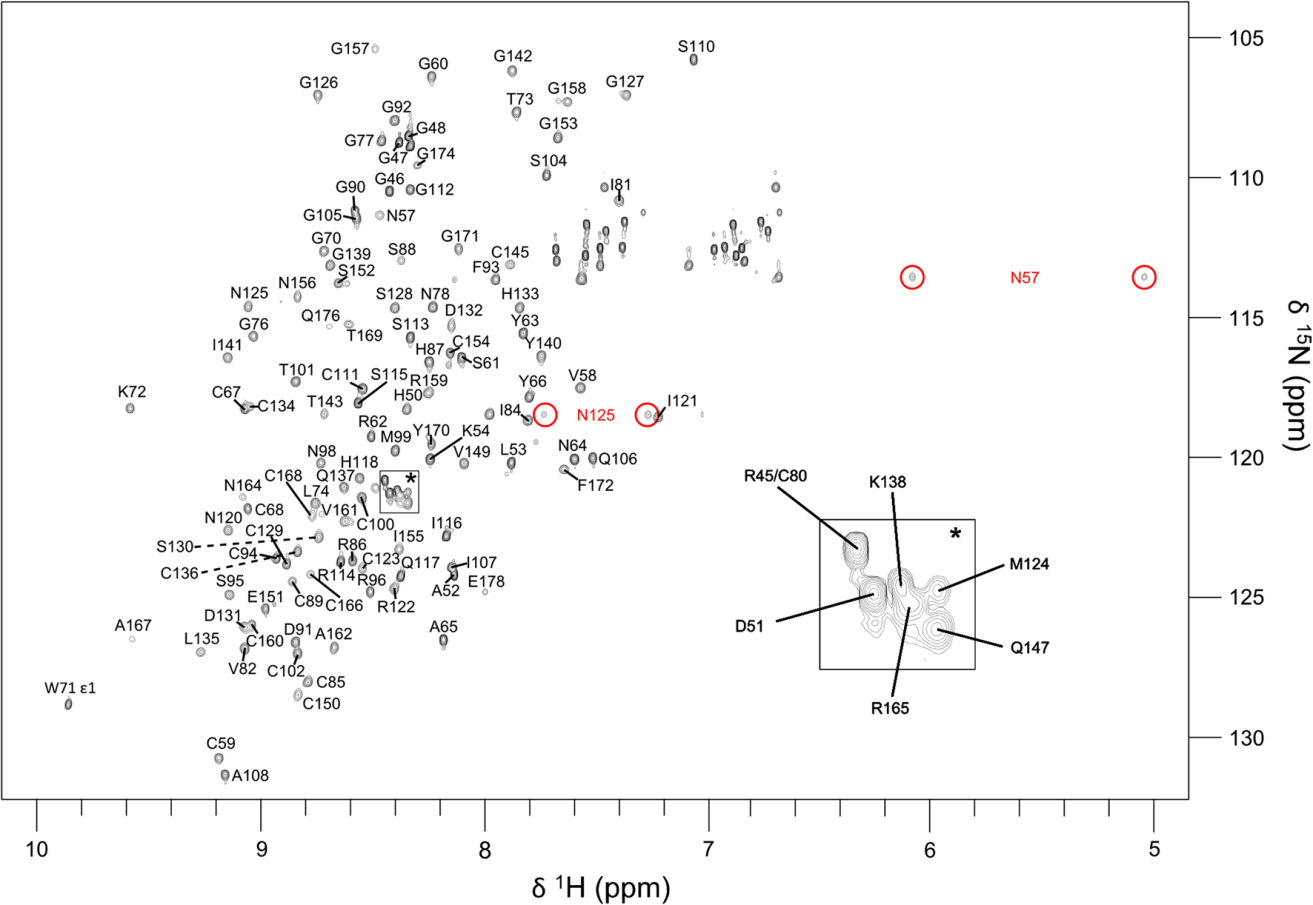
750 MHz ^1^H-^15^N HSQC spectrum of ^15^N-labelled FUN-EGF3 in 5 % D_2_O/95 % H_2_O at pH 5.4 and 298 K. *Peak* assignments for backbone amides and the W71 Nε1-Hε1 *peak* are shown. The NH_2_ side-chain peaks of N57 and N125 are also indicated by *red circles*. Assignments are not shown for overlapping peaks. The *boxed region marked* * is annotated separately on the *lower right-hand side*

Resonances were assigned for all 134 residues in the native sequence, starting at R45. Figure 2 shows a ^1^H-^15^N HSQC spectrum of FUN-EGF3. All non-proline backbone ^1^H^N^ and ^15^N resonances were assigned except C119 and T173. ^15^N assignments of the 10 proline residues were not obtained. Overall, 97.3 % of all ^1^H assignments were obtained, including 100 % of ^1^Hα, 99.0 % of ^1^Hβ, 100 % of ^1^Hγ and 97.6 % of ^1^Hδ. 89.9 % of ^13^C resonance assignments were obtained, including 100 % of ^13^Cα, 99.1 % of ^13^Cβ and 100 % of ^13^C′. Complete proline side-chain ^1^H and ^13^C assignments were obtained. The remaining unassigned ^1^H and ^13^C resonances are mainly in aromatic side chains.

Several unusual chemical shifts were measured, most likely arising from the proximity of these nuclei to aromatic rings. The ^1^Hδ2 side-chain resonances of N57 are upfield-shifted, with chemical shifts of 5.05 and 6.09 ppm (Fig. 2). NOESY data indicate that the side chain of N57 in the FUN domain is close to the W71 aromatic ring, also in the FUN domain (data not shown). The ^1^Hβ resonance of T101 in the EGF1 domain is also upfield-shifted (2.76 ppm)—the atom is likely to lie above the plane of the aromatic ring of F93 (data not shown). In addition, the ^15^Nδ resonance of N125 in the EGF2 domain is downfield-shifted, with a chemical shift of 118.35 ppm (Fig. 2).

**Fig. 3:**
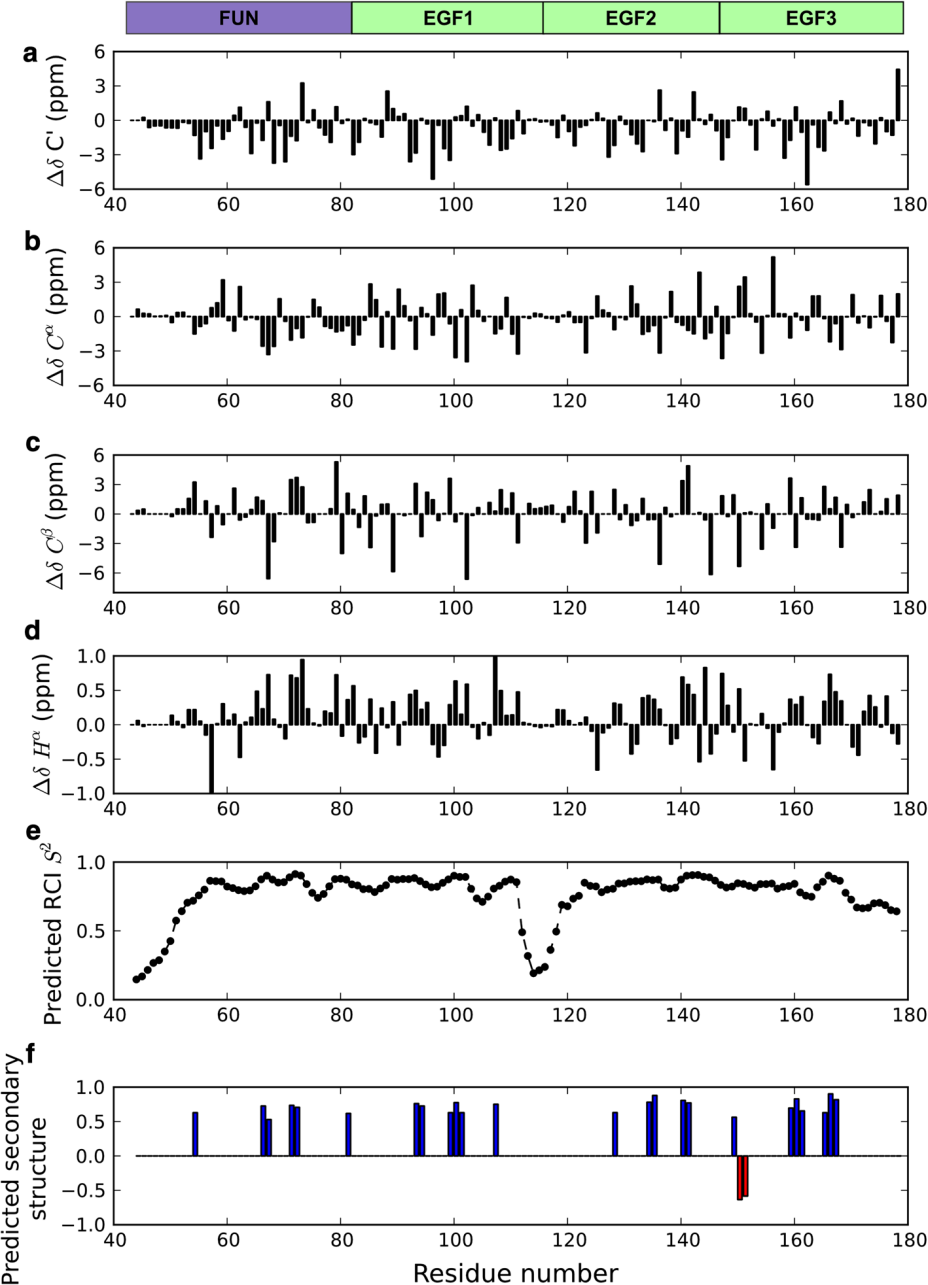
Analysis of FUN-EGF3 chemical shifts. Domains, defined by exon boundaries in the *FBN1* gene (Pereira et al. 1993), are indicated by *coloured boxes* above the plots. **a** ^13^C*′* secondary chemical shifts plotted as a function of residue number. **b** ^13^Cα secondary chemical shifts. **c** ^13^Cβ secondary chemical shifts **d** ^1^Hα secondary chemical shifts. **e** Random coil index (RCI) order parameters (*S* ^2^) predicted from chemical shifts, plotted as a function of residue number. Predictions were made using the TALOS+ and MICS servers (Shen et al. 2009, Shen and Bax 2012), using the method of Berjanskii and Wishart (2005). **f** Secondary structure prediction by TALOS+/MICS. *Bars* indicate the predicted secondary structure adopted by each residue, with the height showing the prediction probability. Positive values and *blue bars* indicate extended (β-strand) structure, while negative values and *red bars* indicate helical (α-helix) structure. Residues without *bars* are predicted to occur in loops, turns or regions with random-coil structure

Secondary chemical shifts (Fig. 3a-d) indicate that the majority of residues are in structured regions of FUN-EGF3, as the values differ from random coil chemical shifts. They were used to make predictions about the structure and dynamics of the protein. Figure 3e shows a plot of predicted order parameters (*S* ^2^), on the basis of the random coil index (Berjanskii and Wishart 2005, Shen et al. 2009, Shen and Bax 2012), against residue number. The predictions identify several potentially flexible regions in the structure: firstly, the N-terminal residues (R45-G55) have predicted *S* ^2^ < 0.8; secondly, residues in the linker sequence between the last cysteine of the EGF1 domain and first cysteine of EGF2 (G112-H118) all have predicted *S* ^2^ < 0.5. Thirdly, residues at the C-terminus of the EGF3 domain, after the penultimate cysteine (T169-E178) may be more flexible, all having predicted *S* ^2^ < 0.8. In the full-length fibrillin molecule this loop may pack against the hyb1 domain, which follows the EGF3 domain in the amino acid sequence. There is also variability in the predicted *S* ^2^ value in other regions of the structure-residues with lower values may lie in flexible loops.

Chemical shift-based predictions by TALOS+ (Shen et al. 2009) and MICS (Shen and Bax 2012) suggest that there are no long, continuous regions of regular secondary structure in FUN-EGF3 (Fig. 3f). However, there are several stretches of two to three residues with predicted β-sheet-like structure. These mainly coincide with the expected locations of β-hairpin structures, which are typically found in EGF-like domains (Bork et al. 1996). For example, residues R159-V161 and R165-A167 in the EGF3 domain are expected to form a two-stranded β-sheet. Only two residues, C150 and E151 in the EGF3 domain, are predicted to adopt α-helical secondary structure. EGF-like domains typically have only small regions of regular secondary structure, but are stabilised by the presence of three disulphide bonds. The FUN domain, which has an unknown structure, is also predicted to contain little secondary structure, with short β-sheet-like elements. It has four cysteine residues, which may form two disulphide bonds. In future, structure determination of the FUN-EGF3 fragment and characterisation of its dynamics will shed further light on the properties on the FUN domain and the interdomain interfaces.

The chemical shift assignments for FUN-EGF3 have been deposited in the BioMagResBank (http://www.bmrb.wisc.edu) under the accession number 18843.
